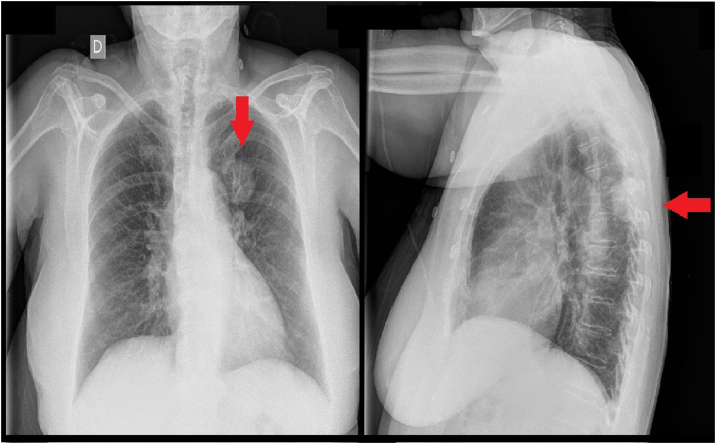# Hamartoma pulmonar incidental en estadificación de neoplasia endometrial

**DOI:** 10.1016/j.aprim.2021.101977

**Published:** 2021-02-16

**Authors:** Jaime Casal Álvarez, Víctor Arenas García, Bárbara Rodríguez Pérez

**Affiliations:** a Servicio de Medicina Interna, Hospital San Agustín, Avilés, Principado de Asturias, España; b Centro de Salud La Magdalena, Área Sanitaria III, SESPA, Avilés, Principado de Asturias, España

## Introducción

El hamartoma es un tumor pulmonar benigno de frecuente localización periférica, más frecuente en varones, entre la quinta y la sexta década de la vida. Suelen ser asintomáticos, y diagnosticados como hallazgo incidental en el estudio de otra enfermedad. Generalmente están formados por cartílago hialino maduro, tejido fibroso, grasa, y en menor medida, vasos sanguíneos. Presentan bordes bien definidos, y en el interior calcificaciones con bordes irregulares dando una imagen característica denominada como «calcificaciones en palomitas de maíz». El tratamiento suele ser quirúrgico si la lesión es accesible, o da clínica al paciente. En caso de ser asintomático y sin signos de malignización en muchos casos se opta por vigilancia radiológica.

## Caso clínico

**Figura 1:**
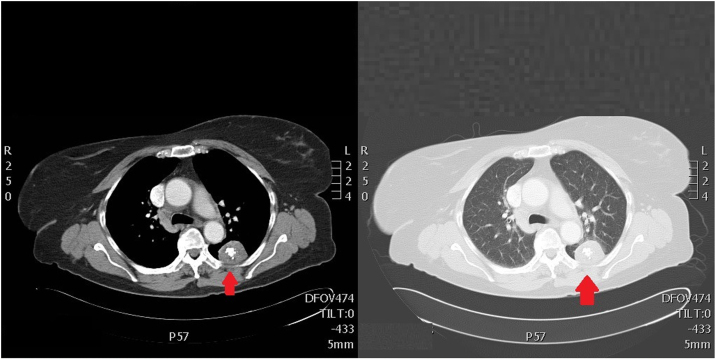

Paciente de 74 años, con reciente diagnóstico de cáncer de endometrio, para cuya estadificación se realizó una TAC toracoabdominal donde se observó una imagen redondeada de unos 3,8 cm de diámetro con calcificación en «palomita de maíz», compatible con un hamartoma (fig. 1). Fue valorada por el servicio de neumología decidiéndose vigilancia radiológica de forma ambulatoria, con revisión en consulta externa cada 18 meses, realizándose radiografías de tórax (fig. 2) seriadas, donde no se objetivó aumento de tamaño de la lesión ni cambios en las características radiográficas de la misma, donde se observa la masa en la región posterior del lóbulo superior izquierdo con contornos perfectamente regulares y ligeras calcificaciones en su interior. Finalmente, tras varios años de seguimiento sin cambios radiográficos ni clínicos fue dada de alta.

**Figura 2:**